# Evaluating the efficacy and safety of the myrtle (*Myrtus communis*) in treatment and prognosis of patients suspected to novel coronavirus disease (COVID-19): study protocol for a randomized controlled trial

**DOI:** 10.1186/s13063-020-04915-w

**Published:** 2020-11-26

**Authors:** Maryam Azimi, Fatemeh Sadat Hasheminasab

**Affiliations:** 1 Gastroenterology and Hepatology Research Center, Kerman University of Medical Sciences, Kerman, Iran; 2 Department of Traditional Medicine, School of Persian Medicine, Kerman University of Medical Sciences, Kerman, Iran; 3 Pharmacology Research Center, Zahedan University of Medical Sciences (ZaUMS), Zahedan, Iran

**Keywords:** COVID-19, Myrtle, Herb, Persian medicine, *M. communis*

## Abstract

**Background:**

Since December 2019, the outbreak of coronavirus pneumonia was observed in China and quickly propagate in all of the world. Nowadays, many trials are underway on this disease in which the efficacy of various therapeutic remedies including chemical or natural agents as well as different non-pharmacological methods such as acupuncture are evaluated. This study aims at investigating the effect of *M. communis* fruit for treatment of COVID-19 disease.

**Methods:**

We are performing an open-label randomized controlled trial on outpatients clinically suspected to COVID-19 disease in the age range of 18–65 years old with mild to moderate symptoms and without respiratory distress. Patients in both groups (*M. communis* and control) receive conventional therapy, but those in *M. communis* group get *M. communis* preparation in addition to conventional therapy. Intervention will continue for 5 days and the study outcomes including clinical status as well as mortality rate and adverse effects will be measured up to 14 days.

**Discussion:**

The protocol describes the design of an ongoing randomized controlled trial to establish the evidence for the usage of water extract of *M. communis* fruit in clinically suspected COVID-19 disease and identify any safety concerns.

**Trial registration:**

The trial has been registered at the Iranian Registry of Clinical Trials website under the code IRCT20180923041093N3 on March 28th, 2020 (https://www.irct.ir/trial/46721). The results will be disseminated through manuscript publications and presentations to scientific meetings.

## Background

The outbreak of the pneumonia caused by the novel coronavirus (COVID-19) was first observed in Wuhan, Hubei province, China, in December 2019 and quickly spread all over the country, and then almost all throughout the world, it formed a global concern as a pandemic [1, 2]. The infection caused by the novel coronavirus has a wide range from asymptomatic infectious to mild upper respiratory tract disease which may lead to severe pneumonia and even death [3, 4]. At the onset of clinical symptoms, most patients have such complaints as fever, cough, shortness of breath, muscular pain, and fatigue. Some patients also experience loss of taste and smell, headaches, or diarrhea. Patients with mild symptoms may have only fever and fatigue, whereas in severe cases, patients experience shortness of breath, hypoxia, and acute respiratory distress syndrome, which may include severe metabolic acidosis, coagulation disorders, and septic shock. The mortality rate varies in different age groups and under different conditions and underlying diseases, but it is generally not high compared to similar diseases; nevertheless, due to the high probability of transmission of this disease, mortality and economic costs are significant [5–7].

Healers with different therapeutic approaches, including classic medicine, herbal medicine, acupuncture, Chinese medicine, and Persian medicine have intent to find a solution to cure or lessen the signs and symptoms of this contagious disease [7, 8]. Exploring the clinical trials registered on different World Health Organization Primary Registries proved a notable attention to complementary and alternative medicine (CAM) for controlling the novel coronavirus pneumonia [7]. Previously, considerable studies had been designated in the field of CAM for prevention, treatment, and rehabilitation of Severe acute respiratory syndrome (SARS) and influenza [9].

The aqueous extract of *M. communis* fruit in combination with sugar as a *M. communis* syrup is an ancient remedy for pneumonia recommended in Persian medicine manuscripts. Heart tonic, lung tonic, antitussive, and anti-diarrhea activities are some of the properties mentioned for *M. communis* [10, 11]. Recent studies demonstrated the antioxidant, anti-inflammatory, anti-viral, and anti-microbial properties of this herb. Therefore, considering the pharmacological activities of *M. communis* in addition to traditional approval, this remedy seems to be a promising candidate for performing clinical trials and assessing its efficacy on controlling this disease.

## Methods/design

### Study objectives and hypothesis

The main purpose of the proposed trial is to determine whether *M. communis* preparation can accelerate the healing process of patients clinically suspected to COVID-19 pneumonia and decrease the hospital admission and other related complications.

Primary hypothesis: taking *M. communis* fruit preparation in the first days of clinical suspicion to COVID-19 will subside the sign and symptoms of the disease, as well as decrease the respiratory distress and enhance the well-being.

### Public involvement

The present trial is designed against the uncertainties about the value of applying alternative therapy and herbal medicine for alleviating the symptoms and promoting the prognosis of the mentioned disease. This issue is a popular subject raised by both people and health professionals involving the current pandemic.

### Ethical aspects

The protocol of this study is approved by the Local Medical Ethics Committee of Kerman University of Medical Sciences under the approval code *IR.KMU.REC.1399.015*; it is also registered at the Iranian Registry of Clinical Trials website under the code *IRCT20180923041093N3*. This study will be conducted in accordance with the guidelines of Declaration of Helsinki (2008 revision). The procedure will be explained to the patients complying with the inclusion criteria, and each participant will voluntarily sign an informed written consent.

### Confidentiality

Data and source documents will be archived for the purposes of any need for monitoring or inspection by the Ethics Committee. The researchers are only allowed to publish the general and group results of this research without mentioning patients’ name and details. The Research Ethics Committee can access patients’ information to monitor their rights.

### Patients recruitment

Patients clinically suspected to the COVID-19 pneumonia are considered and enrolled as suspected cases if they meet either an epidemiological history and two clinical manifestations or three clinical manifestations without epidemiological history. Other inclusion criteria include the age range of 18–65 years old, absence of respiratory distress, and candidate for outpatient care and home isolation. They would be eligible if they do not have exclusion criteria including pregnancy, lactation, allergy to *M. communis*, diabetes, hypertension, hepatic disorder, and renal disorder. Exclusion criteria further covered recent consumption of herbal drugs. The criteria for discontinuing the trial are allergy or any other adverse effects to *M. communis*, applying other herbs during the project, and irregular consumption of *M. communis* preparation.

### Study design

This open-label randomized controlled clinical trial will be conducted to determine the effect of *M. communis* preparation on subjects clinically suspected to COVID-19 pneumonia. In this trial, the allocation ratio was considered 1:1.

### Study setting

This study will be conducted in the referral clinic of Afzalipour Hospital affiliated to Kerman University of Medical Sciences, Kerman, southeastern Iran. A trained general physician will visit the patients. Next, in case of clinical diagnosis of COVID-19 pneumonia, they will be introduced to researchers. Finally, patients who meet eligibility criteria will be invited to the study. Patients’ recruitment has been started from April 2, 2020.

### Randomization and allocation

All eligible patients will be randomly allocated to intervention and control groups. A biostatistician generated a randomization list via a blocked randomization method (non-stratified, four patients in each block) using Microsoft Excel® software. A secretary enrolls participants to intervention groups via sequentially numbered opaque envelopes.

### Intervention

The researchers obtain written informed consent from the participants, then they are randomly divided into intervention (*M. communis*) and control groups. Patients in both groups are permitted to take only conventional treatment which are indicated in the fifth edition of the novel coronavirus pneumonia guideline of the Iranian Ministry of Health and Medical Education. According to this guideline, the allowed medication for outpatients who are not high risk is supportive care such as acetaminophen, and for those who are high risk is hydroxyl chloroquin sulfate or chloroquine phosphate. It should be noted that high-risk patients are excluded from this study. Patients in the intervention group should take *M. communis* preparation in addition to classic medication. So, they received packets containing *M. communis* fruit and sugar. On a daily basis, they should gently boil the contents of a pack containing 10 g of *M. communis* fruit and 10 g of sugar in 3 glasses of water until 2 glasses of the liquid remain; next, they should percolate it and drink 1 glass in the morning and 1 glass in the evening for 5 days.

Trained assessors collect data and record them in prepared forms. The compliance of the participants will be evaluated via a telephone survey to record the usage of study medication and any side effects. A team from the Vice-Chancellor for Research monitors the processes. The test schedule and procedures are provided in Table 1.

**Table 1:** The test schedule and procedures of suspected COVID-19 patients participating the study

Study phase	Screening	Randomization/intervention phase					Follow-up	
Study days	Day 0	Day 1	Day 2	Day 3	Day 4	Day 5	Day 7	Day 14
Informed consent	x							
Assessing the eligibility	x							
Demographics	x							
History and Physical examination	x							
Assessing the variables	x	x	x	x	x	x	x	x
Pulse oximetery	x							
Randomization		x						
Recording the adverse effect			x	x	x	x		

### Ancillary and post-trial care

Patients are followed up directly for 9 days after the intervention. They can contact the researchers until a month later in case of any presenting adverse effects. Researchers are responsible for providing treatment conditions to eliminate any side effects caused by the intervention.

### Outcome measures

The outcomes will be determined in different time points including 0, 1, 2, 3, 4, 7, and 14 days after the intervention, and the primary assessment time will be on the 7th day after the intervention.

The primary outcome and the method of measurement is as follows:

- Cough (severity), via Fisman Cough Severity Score, graded as five points (0, 1, 2, 3, 4) varying between 0 (no cough) and 4 (severe cough with chest discomfort) [12]

The secondary outcomes include the following:

- Temperature, via thermometer (centigrade)
- Myalgia, via visual analog scale (VAS) [13]
- Weakness, via VAS
- Cough (frequency), via Fisman Cough Severity Score
- Respiratory rate (the number of breaths per minute)
- Hospital admission (%)
- Taste and smell disorder (%)
- Mortality rate (%)
- Adverse effect

### Sample size

Due to the lack of a similar study, the primary sample size was initially considered to be 70 (35 in each arm) [14]. Then an interim analysis will be done and the final sample size will be calculated with the comparison of parameters (mean ± standard deviation of cough severity) between the intervention groups on the 7th day after intervention, using the following formula.

$$ n=\frac{{\left({Z}_{1-\alpha /2}+{Z}_{1-\beta}\right)}^2\left({S}_1^2+{S}_2^2\right)}{{\left({\mu}_1-{\mu}_2\right)}^2} $$

Type I (α) and type II (β) errors will be set at 0.05 and 0.1, respectively.

If the primary sample size is adequate, the study will be finished, and if it is inadequate, the study will be continued (under the supervision of the ethics committee of Kerman University of Medical Sciences).

### Statistical analysis

Only the data of participants who complete the follow-up will be considered. Their demographic data including gender and age will compare between the two groups using the chi-square test. To compare the changes in the symptoms experienced by the patients in the two groups at 7 different time points (on enrollment, 1, 2, 3, 4, 7, and 14 days), the repeated measures ANOVA will apply. The independent samples t-test will also be utilized for comparing the changes between the two groups. Differences of groups (*M. communis* and control) will be reported as mean and 95% confidence intervals. Thus, the resultant value of *p* < 0.05 will be considered significant. The statistical analysis will perform via SPSS 23.

### Definition of end of the study

The end of study will be the last patient’s last visit. However, the Vice-Chancellor for Research and ethics committee of Kerman University of Medical Sciences have supervision on trial and make the final decision to terminate the study.

### Data management

There are no legal, ethical, or security issues related to recording, collection of the data, storage, processing, and dissemination for this trial. We will not generate any sensitive data, and also we will not undersign any confidentiality contract. All data will be archived for up to 10 years after the study.

### Potential weakness in study design

The protocol of this study was conceived when the PCR test was not adequately available for confirming the diagnosis of COVID-19 infection; on the other hand, the placebo-controlled setting can enhance the value of the study. Hence, it can be concluded that another placebo-controlled study on the confirmed COVID-19 patients is required and ethically justifiable.

Lack of monitoring such laboratory data as inflammatory factors, lymphocyte count, and renal and liver function, as well as the lack of following the chest radiography, are the other limitations of this study.

## Discussion

The efficacy, safety, and availability of the treatment are the key factors indicating the success of any drug in being welcomed. Previous successful experiences on the efficacy of herbs of traditional Chinese medicine in managing SARS, middle east respiratory syndrome (MERS), and influenza have resulted in designing various researches on different aspects and capacities of alternative medicine for alleviating COVID-19 disease [8]. The present research will provide evidence as to whether *M. communis* is safe and appropriate for treating COVID-19 pneumonia. *Myrtus communis*, as a potent anti-viral agent, may be useful especially in the early stage of the disease [15]; on the other hand, its anti-inflammatory property can reduce the cytokine storm [16]. The efficacy of this herb on diarrhea has been proved in several researches [17]. In addition, based on the ancient Persian medicine resources, *M. communis* extract can be recommended for pneumonia especially when accompanied by cough and diarrhea [11].

The evaluation of the safety of the plant is very crucial especially in such significant projects as COVID-19 disease. Previous clinical trials on *M. communis* did not mention any serious adverse effects [18]. On the other hand, *M. communis* is used only for 5 days. Therefore, there are no major concerns regarding the possible side effects of a long-term consumption. In addition, the growing trend of this disease, its high costs of treatment and hospitalization, and resource constraints prove the need to explore safe, effective, and inexpensive COVID-19 medications for shortening the disease course and enhancing the prognosis. Hence, an evidence-based clinical trial to evaluate the effectiveness of *M. communis* in treating pneumonia induced by COVID-19 certainly has merit.

Resultantly, we have described a clinical trial for the treatment of COVID-19 pneumonia using *M. communis* in Kerman, Iran. Moreover, based on the results of this study, we will hold a large-scale clinical trial, with the aim to comprehensively assess the efficacy and safety of *M. communis* against COVID-19 infection.

## Trial status

The protocol version number is two with revision ID: *137273* and registration date: June 3, 2020. The patient recruitment for this research has begun on April 18, 2020. It is expected to continue till July 30, 2020. Any modification in coordination with the Ethics Committee will be recorded on Iranian registry of clinical trials web-site.

## Data Availability

The results of this study have the potential for public health applicability. The target audience will be reached through oral presentations, publications, and seminars. The researchers will have to be sure that the participant’s privacy is maintained. Data and source documents will be archived for the purposes of any need for monitoring or inspection by the Ethics Committee. At the end of the study, participants will be able to access a copy of the results of the trial from the researchers.
